# Detection of novel viruses in porcine fecal samples from China

**DOI:** 10.1186/1743-422X-10-39

**Published:** 2013-01-30

**Authors:** Jie-mei Yu, Jin-song Li, Yuan-yun Ao, Zhao-jun Duan

**Affiliations:** 1 National Institute for Viral Disease Control and Prevention, China CDC, 100052, Beijing, China

**Keywords:** 454 Pyrosequencing, Astroviruses, Bocavirus, Parechovirus

## Abstract

**Background:**

Pigs are well known source of human infectious disease. To better understand the spectrum of viruses present in pigs, we utilized the 454 Life Sciences GS-FLX high-throughput sequencing platform to sequence stool samples from healthy pigs.

**Findings:**

Total nucleic acid was extracted from stool samples of healthy piglets and randomly amplified. The amplified materials were pooled and processed using a high-throughput pyrosequencing technique. The raw sequences were deconvoluted on the basis of the barcode and then processed through a standardized bioinformatics pipeline. The unique reads (348, 70 and 13) had limited similarity to known astroviruses, bocaviruses and parechoviruses. Specific primers were synthesized to assess the prevalence of the viruses in healthy piglets. Our results indicate extremely high rates of positivity.

**Conclusions:**

Several novel astroviruses, bocaviruses and Ljungan-like viruses were identified in stool samples from healthy pigs. The rates of isolation for the new viruses were high. The high detection rate, diverse sequences and categories indicate that pigs are well-established reservoirs for and likely sources of different enteric viruses.

## The study

Many emerging viruses are of zoonotic origin and can cause epidemics in humans after overcoming the inter-species barrier through mutation or other genetic events, including recombination. The identification of previously unknown viruses in animal hosts is important for understanding their potential for cross-species transmission and emergence. Pigs are known to harbor a diverse array of viruses, including coronaviruses, astroviruses and kobuviruses [1-4], among which coronaviruses and astroviruses are well known viruses that can infect human. To better understand the spectrum of viruses present in pigs, we utilized the 454 Life Sciences GS-FLX high-throughput sequencing platform (454 Life Sciences/Roche, Branford, CT, USA), which has emerged as a non-biased, comprehensive and powerful tool for virus discovery in complex environmental samples [5,6], to sequence stool samples from healthy pigs.

With the cooperation of the Lulong County CDC, stool samples were collected from healthy piglets < 3 months of age from several farms and sporadically distributed families that raised pigs in Lulong County during 2006–2009. The samples were transported on dry ice and stored at −80°C at the Chinese CDC.

The samples were screened for rotavirus, calicivirus and astrovirus by ELISA or routine PCR. Nine of the samples negative for these viruses were pooled, diluted (1:5 ratio, wt/vol) and then sequentially filtered through 0.45- and 0.22-μm membranes. The pooled sample included two samples that were previously demonstrated to contain a novel porcine bocavirus (6V/7V CHN) that was partially sequenced in our lab [7]. Total nucleic acid was extracted from the pooled sample and cDNA was generated using random hexamers. Random PCR amplification [8] was performed and the amplified cDNA was used as the template for standard library construction and sequencing using Roche Genome Sequencer FLX Titanium pyrosequencing technology.

**Figure 1:**
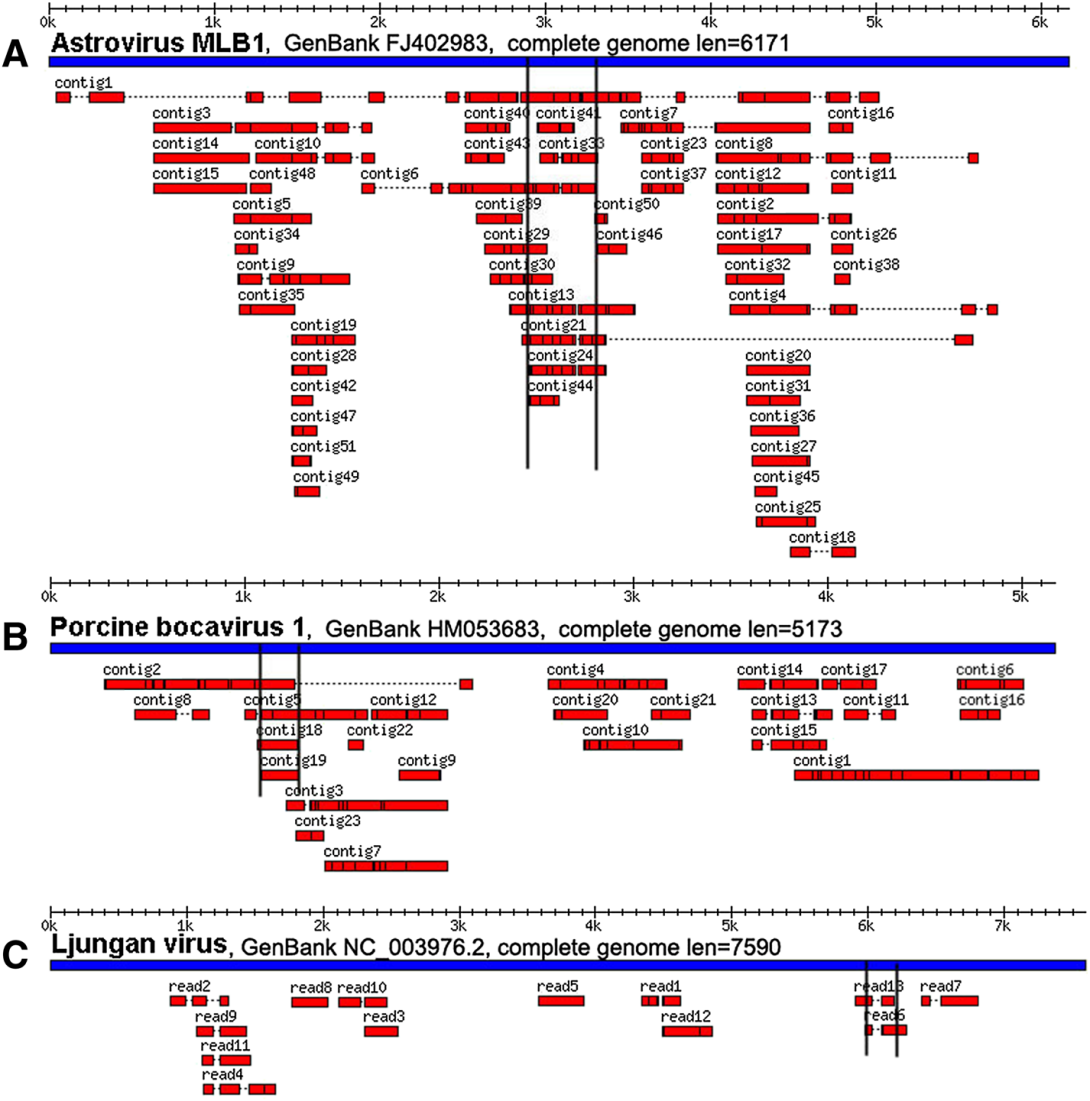
**Sequences sharing similarity with astrovirus MLB1, porcine bocavirus 1 and Ljungan virus.** Viral sequences were positionally mapped to the complete genome of (**A**) astrovirus MLB1 (GenBank FJ402983), (**B**) porcine bocavirus 1 (GenBank HM053693) and (**C**) Ljungan virus (GenBank NC_003976.2) based on the similarities of the associated amino acid sequences to the corresponding reference genome proteins. Contig 22 of astrovirus exhibited the highest homology with the capsid protein of porcine astrovirus PAstV-2 (ADP21511.1) but no detectable homology with astrovirus MLB1; therefore, this is not shown on the map. Blue bar: reference genome; red bar: viral sequences that share homology with the reference genome; dotted line: viral sequences that have no homology to the reference genome; vertical line: sequences between the vertical lines were used in the phylogenetic analysis.

The initial pyrosequencing runs produced in excess of 145,000,000 bases of high-quality nucleotide sequence with an average read length of 360 bp. We used a customized informatics pipeline as described previously [9] with minor modifications to computationally identify viral sequences. Briefly, raw sequence reads were filtered to remove low-quality and repetitive sequences; BLASTn and BLASTx were used to identify sequences with similarity to known viruses in GenBank, then sequences identified as viral were further classified into viral families based on the taxonomy of the best hit. Sequence assembly was performed using Newbler (454 Life Sciences) with parameter be set as 90% nucleotide identity over 50 base pairs. Based on this analysis, 348, 70 and 13 unique reads had limited similarity to known astroviruses, bocaviruses and parechoviruses, respectively. Assembly of the raw reads for astrovirus and bocavirus generated 51 and 23 contigs, respectively. Due to the limited number of reads with similarity to parechoviruses, individual reads rather than contigs were analyzed. Most of the sequences detected were highly divergent compared to the most closely related viruses (Figure 1 and Additional file 1: Table S1, Additional file 2: Table S2 and Additional file 3: Table S3 in the additional file). The reference genomes were selected to represent the top BLAST match for most of the sequences.

**Figure 2:**
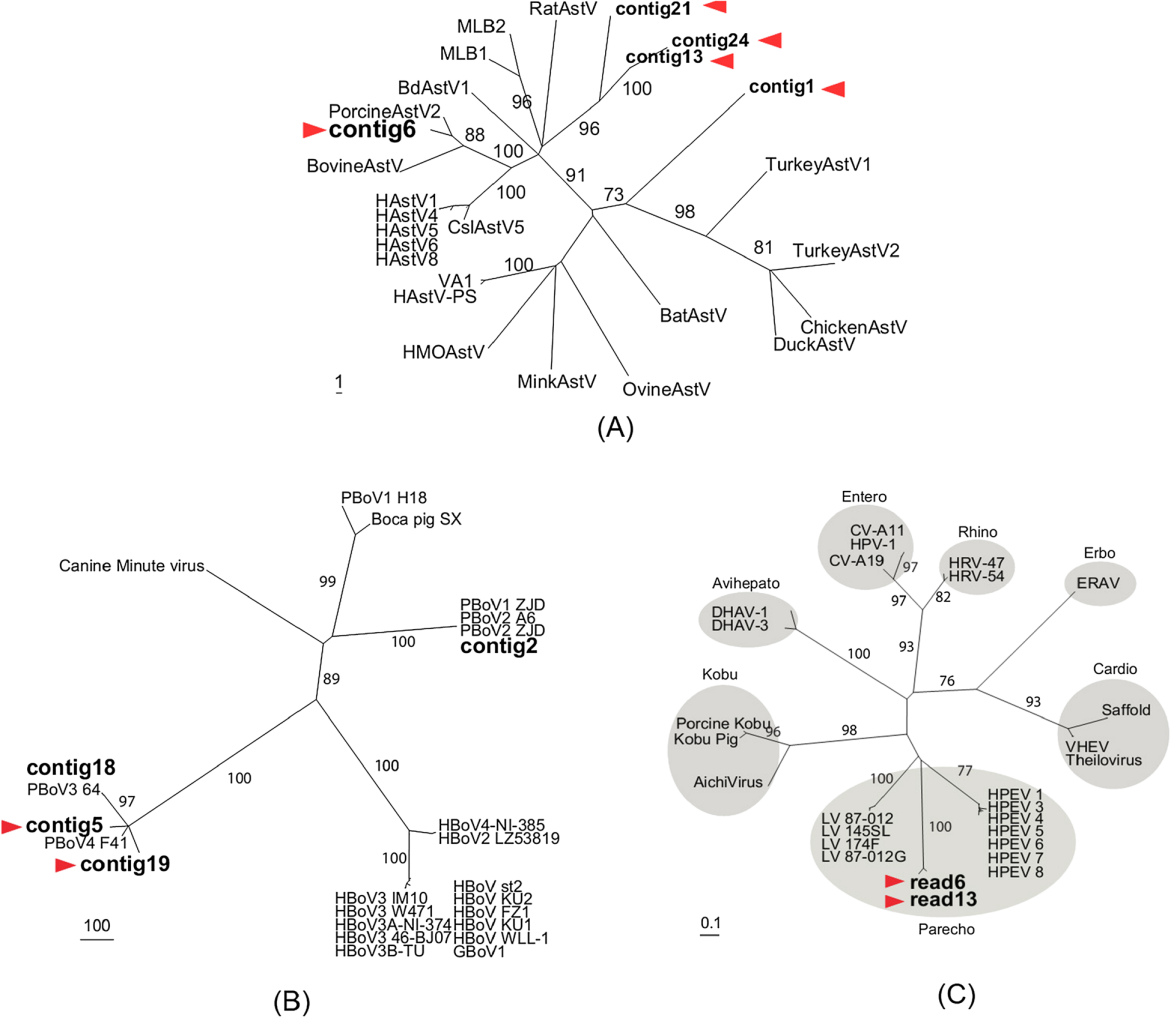
**Phylogenetic analysis of nucleotide sequences of the potentially novel viruses: (A) astrovirus, (B) bocavirus and (C) parechovirus.** The tree was constructed using both the maximum likelihood and maximum parsimony methods in the PHYLIP package with 100 bootstrap replicates. Potentially novel viruses are indicated in bold. Bootstrap values are shown on the branches.

*Astroviruses* have been isolated from a number of host species, including humans, minks, sheep, pigs, chicken, ducks, turkeys [10-12] and more recently from marine mammals, dogs, cheetahs and bats [13-16]. They are generally associated with enteric diseases such as diarrhea and vomiting in a number of mammalian species [17]. The most abundant viral reads in this sequencing library were those from astroviruses. The size of the astrovirus contigs generated ranged from 150 to 5674 bp. These contigs shared 30.1–100% amino acid identity, typically over a small region, with the best hits in the NCBI nr database, which suggests that the sequences are highly diverged from those of known viruses. Interestingly, multiple contigs mapped to the same region of the reference genome (GenBank FJ402983), suggesting the presence of multiple variants of astroviruses in the samples. For example, contigs 1, 6, 13, 21 and 24 mapped to the same subregion of ORF1b. A phylogenetic analysis of these five contigs was performed using both maximum likelihood and maximum parsimony methods in the PHYLIP package with 100 bootstrap replicates. The tree demonstrated that contig 1 was most closely related to avian astroviruses, contig 6 was closely related to porcine astrovirus and contigs 13, 21 and 24 formed a separate branch that was distantly related to all the other astroviruses (Figure 2a). The observed diversity suggests the existence of novel astroviruses in pigs.

Human bocavirus (HBoV) was first identified in respiratory samples from patients with respiratory infections in 2005 [18]. Subsequently, various bocaviruses have been found in respiratory and fecal specimens of human or animal origin, including HBoV 2–4, Gorilla bocavirus (GBoV1) and porcine bocavirus (PBoV1–4, 6V, and 7V) [19,20]. The sizes of the 23 bocavirus contigs in this study ranged from 147 to 1256 bp, and they shared 74.6–99.2% nucleotide sequence identities with the best hit in the NCBI nt database. Contigs 2, 5, 18 and 19 mapped to the same portion of the NS1 region of the reference genome but shared less than 80% identity among themselves at the nucleotide level. A phylogenetic analysis of partial NS1 sequences showed that contig 2 was closely related to porcine bocavirus 1–2 while contig 18 was closely related to PBoV3 [20]. Contigs 5 and 19 shared 74.6 and 80.0% nucleotide identities with PBoV4, respectively (Figure 2b). When we compared the contigs to the sequences from porcine bocavirus 6V and 7V, one and two contigs, respectively, exhibited high nucleotide homologies (> 99%) indicating that we were able to recover these viruses once again with our methods. According to the most recent International Committee on Taxonomy of Viruses species demarcation criteria for the genus *Bocavirus* (at least 5% nucleotide sequence divergence in their nonstructural gene), our data indicate the existence of multiple new *Bocavirus* species in pigs.

*Parechovirus* is a genus in the family *Picornaviridae* that comprises two species: human parechovirus (HPEV) and Ljungan virus (LV). HPEV causes mild gastrointestinal or respiratory illness, and has been implicated in cases of myocarditis and encephalitis [21]. LV can cause significant morbidity and mortality in wild rodents as well as in laboratory animals, and it is a suspected human pathogen [22]. Bank voles infected with LV in captivity develop several different pathologic signs and symptoms, including myocarditis, diabetes and encephalitis [23]. Thirteen unique viral reads with similarities to both HPEV and LV were identified in this study. They spanned the LV reference genome (GenBank NC_003976.2), to which the amino acid identities ranged from 22.3 to 55.8%. Based on the sequences, we infer the existence of at least two parecho-like viruses. Reads 4, 9 and 11 belong to one virus, and overlap with read 2 over 190–231 bp but with only 81.1–82.7% nucleotide identity. Although each of the read pairs 1 and 12, 3 and 10 and 6 and 13 overlap when aligned with a common reference genome, the two reads in each pair share less than 50% nucleotide identity. Phylogenetic analyses using partial 3C region sequences demonstrated that reads 6 and 13 form a distinct branch in the *Parechovirus* genus (Figure 2c). It is worthy to mention that these divergent sequences represent some of the first parecho-like viruses reported in pigs till now.

The three types of novel viruses were investigated further. According to the target viruses, five sets of primers were designed and synthesized based on the contigs containing the most reads for each viral type (primers A–E in the Table 1). Positive and negative controls were included in every PCR run and all products were sequenced. An additional five sets of primers (primer 2ndA–E in the Table 1), which targeted internal regions amplified in the 1^st^ round of PCR, were designed and used in a 2^nd^ round of PCR to exclude potential contamination, and the products were sequenced (data not shown). All of the sequences are provided in Additional file 4: S4. The results of the 2^nd^ round of PCR validated all of the samples identified as positive in the 1^st^ round of PCR. Of the 296 samples, 105 (35.5%), 48 (16.0%), 71 (24.0%), 142 (48.0%) and 40 (13.5%) were positive for primers A, B, C, D and E, respectively. Most of the sequences detected through PCR were highly similar at the nucleotide level to astrovirus (> 80% identity), bocavirus (> 80% identity), and parechovirus (> 90% identity) contigs. There were 41 astrovirus sequences < 80% identical to the original contigs, whereas only two bocaviruses sequences were below this threshold. For parechoviruses, only five sequences showed 85-86% identity to the original contigs. When sequences divergent to the contigs were compared with the NCBI database, some were more similar to known sequences while the best hits of others remained with the contigs. Co-infections with both types of the same virus and different viruses were detected. For example, 35 samples were positive for sets A and B (astroviruses) and 47 samples were positive for sets C and D (bocaviruses), while four samples were positive for all five sets of primers. These results indicate the great diversity of the novel viruses and their high detection rates in the samples. Sequences with the length more than 200 bp were submitted to the Genbank under accession number of JX560993-JX561090.]

**Table 1:** **List of primers used in both the 1**^**st **^**and 2**^**nd **^**rounds of PCR in this study**

**Virus**	**Targeted contig/read**	**Targeted region**	**Primer name**	**PCR round**	**Primer sequence**	**Size (bp)**
Astrovirus	Contig 15	Astroviruses ORF1a region	Set A-F	1^st^	5′- STTRCCWTGGSTYTGGGAGAT −3′	402
			Set A-R		5′- ATMAGCCTCCAGCAGAAGCA −3′	
	Contig 3		2ndA-F	2^nd^	5′- ACTGCYTCTACYTWGCAGCAG −3′	199
			2ndA-R		5′- ACATCWGCCARGAACATACC −3′	
	Contig 14	Astroviruses ORF1a region	Set B-F	1^st^	5′- AGGCTAAACCTCAAGTTAG −3′	360
			Set B-R		5′- CCTACAGGAAAAGTTACTC −3′	
			2ndB-F	2^nd^	5′- CACGGATCTCAATGACATGACC −3′	316
			2ndB-R		5′- GCTATTGCACCTATTCAGAC −3′	
Bocavirus	Contig 5	Bocaviruses NS1 region	Set C-F	1^st^	5′- TCTGCCTSAGGTSRGTGAGAA −3′	224
			Set C-R		5′- KATGCATCTCGAGMAYCTCKT −3′	
	Contig 18		2ndC-F	2^nd^	5′- GAGAAYTCKCTGGCTAGACAGA −3′	185
			2ndC-R		5′- AYCTCKTCKAGCGTYCGAGA −3′	
	Contig 19	Bocaviruses NS1 region	Set D-F	1^st^	5′- TCTCTAGCTAGCGAGTTCTC −3′	206
			Set D-R		5′- TAGATGAGGTGCTCGAGATGCA −3′	
			2ndD-F	2^nd^	5′- ACCTCATCTAGCGTTCGAGA −3′	159
			2ndD-R		5′- GCCTGCTAAGATCACCAAA −3′	
LV-like virus	Read 12	LV-like virus 2C region	Set E-F	1^st^	5′- CTCTTCTGCTATGGAACTGCT −3′	452
			Set E-R		5′- TTGCCGAAGTATCAGGCTTC −3′	
			2ndE-R	2^nd^	5′- CTGCTTGGCAATGATGTGTCGA −3′	404
			2ndE-F		5′- ACAAACGTGTGCAGTTTCTGG −3′	

In this study, we demonstrated the existence of different novel astroviruses, bocaviruses and parechovirus in porcine feces using 454 massively-parallel pyrosequencing. These viruses were highly prevalent in healthy pigs < 30 days old. The great diversity and high detection rate of these viruses in the porcine samples indicate that pigs are well-established reservoirs for many different viruses. Human astroviruses, bocaviruses and parechovirus are well-known pathogens that cause a variety of human diseases. It is unknown if the novel viruses detected in this study are pathogenic or have zoonotic potential. Given the diversity of viruses detected in this preliminary study, more attention should be paid to the viromes of pigs and other agricultural animals due to the potential for transmission of these viruses to humans in close proximity to these animals.

## Competing interest

The authors declare that they have no competing interests.

## Authors’ contributions

JM carried out the data summarizing, participated in the 454 high-through out sequencing, study designing and drafted the manuscript. JS participate in the 454 high-through out sequencing. YY carried out the screening of the viruses in the porcine fecal samples. ZJ conceived of the study, and participated in its design. All authors read and approved the final manuscript.

## Supplementary Material

Additional file 1: Table S1 Best hits in terms of nr, percent similarities and e values for the contigs. S1: astroviruses. Click here for file

Additional file 2: Table S2 Bocaviruses. Click here for file

Additional file 3: Table S3 Parecho-like virus. Click here for file

Additional file 4: S4 Sequences of the 1^st^ and 2^nd^ round PCR products of potentially novel viruses from the positive samples. “-” indicates gaps in the sequences. Click here for file
